# Supplementary figures and images for: Highly frequent undesired insertional mutagenesis during Drosophila genome editing
Source: PLoS Genet. 2026 Jul 9;22(7):e1012192. doi: 10.1371/journal.pgen.1012192 (PMC13405110; doi:10.1371/journal.pgen.1012192)

# S1\_Raw\_images

Fig 2B

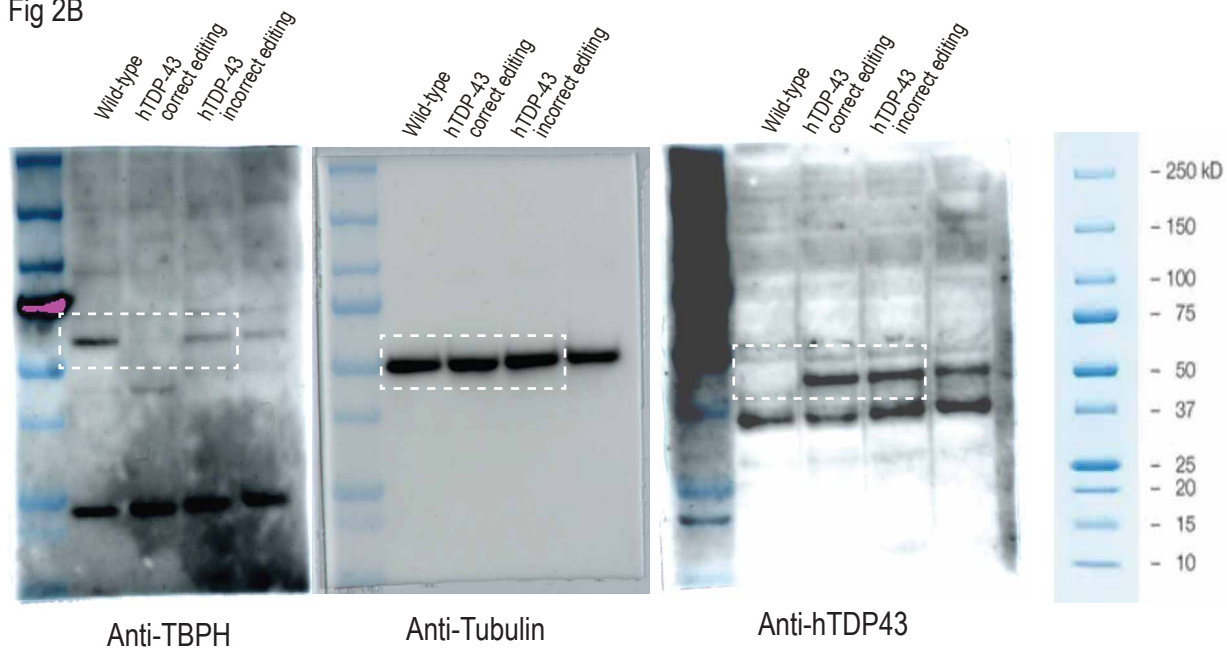

Fig 2D

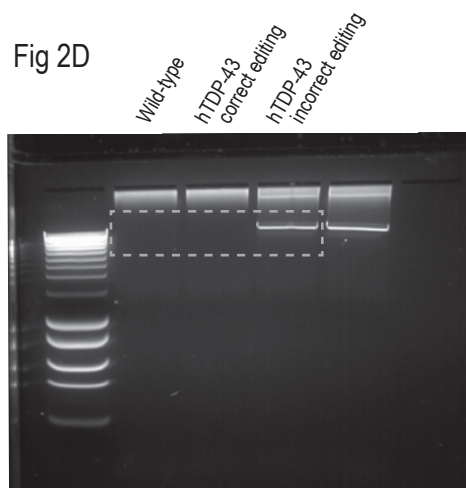

Fig 2F

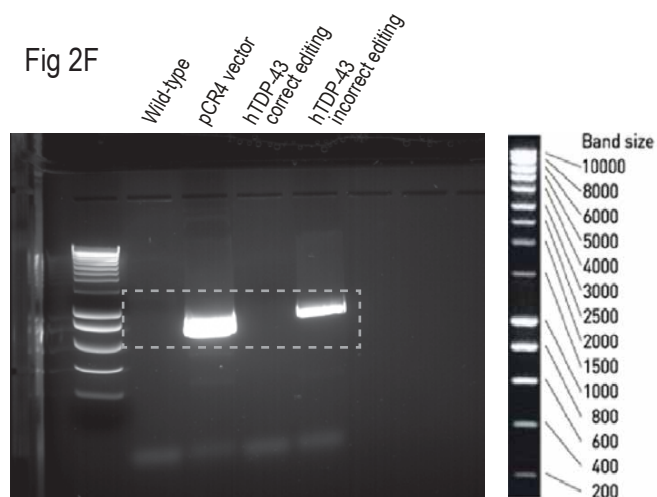

Fig 3C

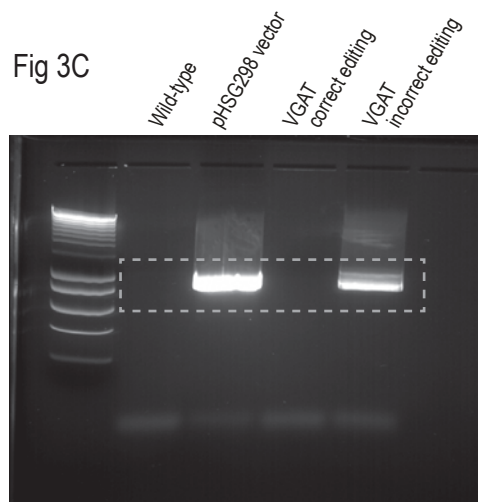

Fig 4C

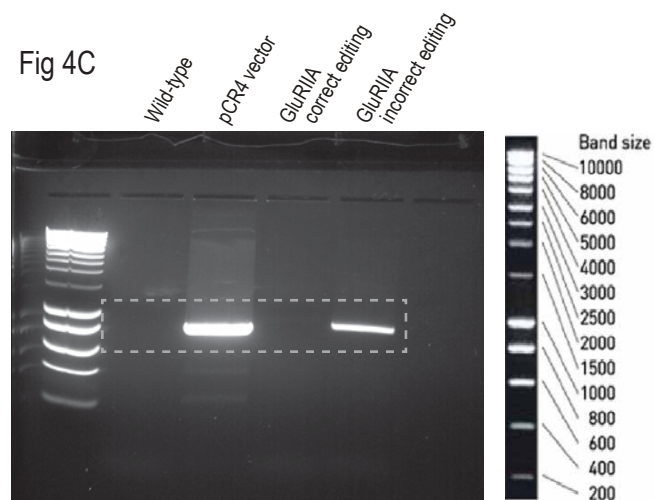

S1\_Raw\_images

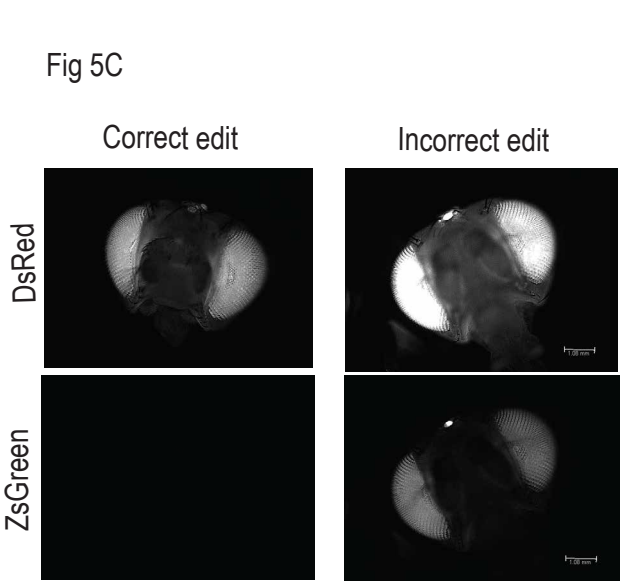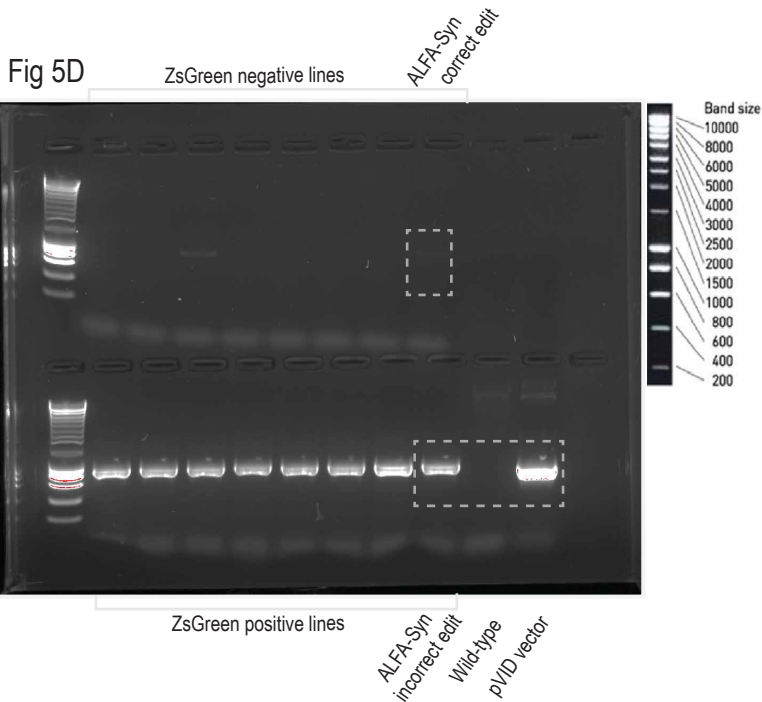

Supplement: S1 File — Fig 2. Undesired genomic insertion of HDR template vector sequence during gene replacement HDR. (B) Western blot showing the expression of hTDP-43, Drosophila TBPH and tubulin loading control in protein extracts derived from Drosophila controls, correctly edited humanised hTDP-43 Drosophila, and a humanised hTDP-43 Drosophila line with incorrect editing due to template genomic insertion. The molecular weight marker (Blue Precision Plus, Bio-Rad) used to identify protein size is shown to the right of the Western blot images (D) Image of an agarose gel from a genomic DNA PCR using a forward primer in the TBPH 3’ end and a reverse primer in the hTDP-43 5’ end, which produces a product of ∼10 kb from genomic DNA derived from incorrectly edited hTDP-43 Drosophila, which is not present in genomic DNA from Drosophila controls or a correctly edited replacement hTDP-43 line. (F) Image of an agarose gel from genomic DNA amplification using primers specific for the template pCR4 vector. Vector plasmid sequences can be detected in vector DNA and genomic DNA derived from incorrectly edited hTDP-43 lines, but not in genomic DNA derived from correctly edited hTDP-43 Drosophila or controls. Fig 3. Undesired template vector integration in genome editing to insert an epitope tag. (C) Image of an agarose gel of products amplified using PCR using primers specific for the vector pHSG298. pHSG298 sequences can be amplified from pHSG298 and genomic DNA derived from unwanted editing of VGAT Drosophila lines, but not correctly edited VGAT Drosophila lines or controls. Fig 4. Undesired HDR vector genomic integration in genome editing to alter single nucleotides. (C) Agarose gel image of genomic DNA PCR amplification using primers specific for pCR4. The vector backbone sequence was identified in incorrectly edited GluRIIA Drosophila lines, but not in the correctly edited GluRIIA Drosophila line or controls. Fig 5. The pVID HDR template vector enables the identification of Drosophila genome ed [file pgen.1012192.s001.pdf]
