# Supplementary material for: Highly frequent undesired insertional mutagenesis during Drosophila genome editing
Source: PLoS Genet. 2026 Jul 9;22(7):e1012192. doi: 10.1371/journal.pgen.1012192 (PMC13405110; doi:10.1371/journal.pgen.1012192)
Supplement: Attachment — Submitted filename: 160647_1_rebuttal_3731002_tdnbz4.pdf [file pgen.1012192.s002.pdf]

FULL REVISION PLAN

Review Commons

Manuscript number: RC-2025-03162

Corresponding author(s): Brian D. McCabe

Manuscript title: *Highly frequent undesired insertional mutagenesis during Drosophila genome editing*

1. General Statements

We thank all three reviewers for their careful and constructive evaluation of our manuscript. We are pleased that the reviewers recognised the importance of the work we describe and found the experimental approach sound.

This manuscript reports that undesired insertion of the plasmid backbone, including vector sequences not intended to be part of the genome edit, occurs at high frequency during CRISPR/Cas9-mediated HDR in Drosophila. We document this phenomenon across multiple independent genome editing projects, using three different plasmid backbones and targeting distinct genomic loci, demonstrating that it is not an isolated or project-specific artefact. We further introduce pVID, a new donor vector incorporating a ZsGreen negative selection marker that allows straightforward identification and exclusion of lines carrying undesired insertions, providing a practical solution to avoid this genome editing issue.

In response to the reviewers' comments, we have revised the manuscript to: (i) correct and contextualise prior descriptions of this problem, incorporating the references suggested by Reviewer 2; (ii) add a table summarising gRNA characteristics for all editing projects; (iii) expand the discussion of the underlying DNA repair mechanisms, the potential influence of Cas9 source choice, and the relevance of the findings beyond Drosophila; (iv) confirm the stability of problematic template vector insertions across multiple generations; and (v) improve figure clarity, correct typographical errors, and clarify several passages flagged by the reviewers. All responses are described in detail below.

2. Point-by-Point Description of the Revisions

Reviewer 1

Major Comment 1 — **DNA repair pathways underlying backbone capture**

• I think the authors should discuss potential DNA repair pathways (e.g., NHEJ, MMEJ) underlying plasmid backbone capture in more detail. Did you check for knockouts within your screened transformants? That could provide insight into the underlying mechanisms.

Response: We screened humanized TDP-43 line for *tbph* knockouts, since our aim was to fully knock out the Drosophila gene and insert the human ortholog. However, we did not screen any of the other lines described in the manuscript for indels caused by NHEJ, since the dsRed selection we employed would not enable us to recover lines without insertion events. We hypothesise that one of the two gRNAs used being more inefficient than the other causes a single homologous recombination event and insertion of the vector template. However, the underlying mechanism is still unclear, and could be caused by NHEJ, HDR or a combination of these mechanisms as has previously observed (44). We have expanded on potential mechanisms inducing HDR template vector insertion events in the discussion of the revised manuscript.

Major Comment 2 — gRNA characteristics and design parameters

• It would be important to describe gRNA characteristics and general design parameters (GC content, distance from cut to intended edit, homology arm length) and analyze whether these correlate with correct HDR vs. plasmid insertion. A table summarizing these details could help reveal potential trends.

Response: At the reviewers suggestion, we have added a table (Table 1) describing the all the characteristics of the gRNAs further in the material and method section. Unfortunately though, no commonality was immediately apparent to us.

Major Comment 3 — Single versus dual gRNA strategies

• Did the authors consider exploring whether using a single gRNA reduces backbone insertion frequency compared to dual-gRNA strategies? I understand that two gRNAs are needed for your strategy, but it would be interesting to know whether these outcomes are linked to the dual-gRNA design.

Response: *As stated in the discussion, we theorize that perhaps one of the two gRNAs used in our strategies cuts more efficiently and thereby causes a single homologous recombination event and insertion of the vector template. It is possible that originally using a strategy with only one gRNA could cause less insertion of the vector template, however this may be at the cost of gene editing efficiency. Indeed, when Ge et al (17) compared using one versus two gRNAs to induce HDR, they observed more reliable repair events when two gRNAs were used.*

Major Comment 4 — Stability of backbone insertions across generations

• Did you evaluate whether backbone insertions are stable across generations or prone to rearrangement?

Response: *We did keep several of the lines reported in this paper stably across multiple generations, and we have added this observation to the manuscript*

Major Comment 5 — Broader applicability in non-model organisms and therapeutic settings

• A broader discussion of the potential applications of this approach in non-model insects, mammalian cells, or therapeutic settings where HDR is inefficient would be valuable.

Response: *While we only investigated this effect in the creation of CRISPR/Cas9 Drosophila melanogaster models, it is very possible that this could also affect other model organisms or*

cells. We encourage the use of HDR template negative selection markers in all uses of HDR-mediated CRISPR/Cas9 genome editing.

Major Comment 6 — Cas9 promoter and expression level

• The authors also mentioned using a validated Cas9 line (ref #23). What promoter drives Cas9 expression in this line? Did you consider testing different promoters? Since timing of Cas9 expression can be critical, promoter choice may have influenced the results and should be discussed.

Response: *We used the nos promoter for the expression of Cas9, as this promoter is expressed in germ cells and is known to have better efficiency than the other germline promotor like vasa (port et al 2014). However, it is conceivable that the high Cas9 concentration in this line could induce a higher rate of double stranded breaks and thus template vector insertion. We agree it would be interesting to test other Cas9 sources, though this would likely come at the cost of overall editing efficiency. As we describe, the use of pVID now allows negative selection against HDR template vector insertion even with this Cas9 source. We have expanded upon the potential use of other Cas9 sources in the revised discussion.*

Reviewer 2

Major comments

Minor Comment 1 — Line 38: prior descriptions of backbone insertion in Drosophila

Line 38: "this type of unwanted template vector insertion in the case of Drosophila genome editing has to our knowledge not been previously described." Insertion of vector sequences after CRISPR editing in Drosophila and strategies to mitigate such events have been previously described in multiple studies. The authors need to incorporate these into their manuscript.

https://doi.org/10.1242/bio.20147682, https://doi.org/10.1080/19336934.2020.1832416, https://doi.org/10.1534/g3.116.032557.

Response: *We are very grateful to the reviewer for pointing out these prior observations of vector insertion events of which we were not aware. This prior work has now been fully incorporated and referenced in the revised manuscript, and we have removed this erroneous statement. We feel this manuscript validates and quantifies the extent of HDR template insertion across multiple genome editing strategies and templates plus, with pVID, provides a solution to this vexing problem.*

Minor Comment 2 — Line 79: PAM sequence sentence

I have difficulties understanding the following sentence: Line 79: "At this location, on both sides of the insertion, the PAM sequence of the target region was edited to match the PAM sequence of the template donor plasmid." I assume what is meant here is that in the donor vector the PAM sequence was mutated to prevent recutting, but that means this sequence is no longer a PAM. Please rephrase for added clarity.

Response: *The PAM sequence was indeed edited in the template donor plasmid to prevent re-cutting, and we are referring to this edited version of the PAM sequence in this sentence. We edited this sentence this to clarify that the PAM sequences have been edited.*

Minor Comment 3 — Figure 2: panel D arrangement

In Figure 2 panel D is arranged between panels E and F.

Response: *Thank you for pointing this out. We have corrected this error.*

Minor Comment 4 — Primer positions in figures

In Figure 2 it would be useful to also indicate the position of the primers used in 2d in the schematic in 2e. The same applies to Fig. 3a and 4a.

Response: *We have added the position of the primers in figure 2. Since the primers are targeting the backbone of the plasmid commonly in all projects included in this manuscript, we have chosen to only include one figure of this (figure 2).*

Minor Comment 5 — Lines 89–90: duplicated sentence

Lines 89, 90: Duplication of the same sentence.

Response: *Thank you,* We have corrected this mistake.

Minor Comment 6 — VGAT editing: consecutive editing and sgRNA placement

Editing of the VGAT gene: In this case correct editing and plasmid insertions could be found on the same chromosomes. This might be caused by concatemer formation of repair intermediates (as has been described in multiple systems) or by consecutive editing events. Can you please specify whether the donor vector was designed to prevent consecutive editing? I'm also a bit confused about the locations of the sgRNA target sites according to Fig. 3a. It appears that part of the insertion (i.e. the ALFA tag) was encoded on the homology arm and not between the target sites. While such strategies have been described, they are often avoided as the efficiency of insertion decreases with increasing distance to the cut site. Was it not possible to us a sgRNA better matching the insertion cassette?

Response: *For Vgat genome editing, we followed an existing strategy that has been proven effective, reusing the same gRNAs and overall approach to replace the 9×V5 tag with a 1×ALFA tag (see Ref #28, Certel et al. 2022)*

Minor Comment 7 — Line 133: mini-white marker unreliability

Line 133: Please describe why the mini-white marker was unreliable.

Response: In our first design of the pVID vector, we used mini-white as the negative selection marker. However in a number of white eyed lines, we could still confirm the undesired insertion of the HDR template vector. We speculate that expression of mini-white (which we confirmed was not mutated) was repressed in these lines by an unknown mechanism. Since Nyberg et al. (Ref #35) also proposed using mini-white as a negative vector selection marker, we wanted to mention this problem with mini-white negative selection, though we remain unsure of the exact cause. In any case, the use of exogenous ZsGreen in pVID as described in the manuscript fully resolved the issue allowing reliable detection of template vector insertion events as we describe.

Minor Comment 8 — Line 161: "varying frequency"

Not sure I understand the sentence in line 161: If 54% of lines had vector insertion, what does the "varying frequency" refer to?

Response: We have edited this sentence to clarify that 54% of lines had vector insertion.

Minor Comment 9 — pVID availability in methods

Consider highlighting the availability of pVID also in the methods section that described this plasmid.

Response: This has been added to the methods section.

Reviewer 3

We thank Reviewer 3 for their positive assessment of the manuscript and for confirming that no revisions are required.

3. Description of the Revisions Already Incorporated

The following revisions have already been carried out and are included in the transferred manuscript:

- Line 38 has been revised to accurately reflect prior descriptions of backbone insertion in Drosophila, incorporating the references provided by Reviewer 2 (Yu et al., 2014; Ge et al., 2016).
- Line 79 has been rephrased to clarify the logic of the PAM sequence inference.
- A table summarising gRNA characteristics (GC content, distance from cut site, homology arm length) has been added to the Materials and Methods section.
- Six lines carrying backbone insertions were confirmed to be stable over four consecutive generations; this information has been added to the manuscript.
- A discussion of the nos promoter and its potential influence on Cas9 concentration and vector template integration has been added to the Discussion and Limitations sections.
- A statement acknowledging that the phenomenon may occur in other model organisms or cell lines has been added to the Limitations section.
- Panel arrangement in Figure 2 has been corrected (panel D repositioned).
- Primer positions have been added to the schematic in Figure 2e.
- The duplicated sentence at lines 89-90 has been removed.
- The mini-white marker section has been removed to avoid confusion, with a brief explanatory note added.
- The text at line 161 has been clarified to distinguish between the average insertion frequency (54%) and the varying rates observed across individual projects.
- Availability of pVID has been noted in the relevant Materials and Methods section.

- Typographical and figure legend errors have been corrected throughout.

4. Description of Analyses that Authors Prefer Not to Carry Out

We do not plan to perform new experiments directly comparing single-gRNA versus dual-gRNA strategies in terms of backbone insertion frequency. While this would be informative, it would require substantial additional work beyond the scope of the current study. We have instead discussed this question in the context of existing literature and suggested it as a direction for future investigation in the discussion.

We do not plan to perform immunofluorescence or flow cytometry to directly quantify the proportion of cells carrying backbone insertions, as the current study focuses on germline genome editing in whole animals rather than cell-based assays. The gRNA characterisation table and the multi-generation stability data provide the additional experimental context requested by Reviewer 1 within a feasible scope.

We do not plan to extend the study to other model organisms or cell lines at this stage. The observation that backbone insertion may occur in other systems is discussed in the Limitations section, and we encourage investigators in other systems to test for this phenomenon in their own experimental contexts.
